# Chemical and Biological Properties of Different Romanian Populations of Hyssopus officinalis Correlated via Molecular Docking

**DOI:** 10.3390/plants13223259

**Published:** 2024-11-20

**Authors:** Ilinca Merima Imbrea, Magdalena Osiceanu, Anca Hulea, Mukhtar Adeiza Suleiman, Iuliana Popescu, Doris Floares (Oarga), Emilian Onisan, Alina-Georgeta Neacșu, Cosmin Alin Popescu, Calin Hulea, Georgeta Pop, Simona Niță, Florin Imbrea, Diana Obistioiu

**Affiliations:** 1 Faculty of Engineering and Applied Technologies, University of Life Sciences “King Michael I” from Timisoara, Calea Aradului 119, 300645 Timisoara, Romania; ilinca_imbrea@usvt.ro (I.M.I.); emilian.onisan@usvt.ro (E.O.); 2 Faculty of Agriculture, University of Life Sciences “King Michael I” from Timisoara, Calea Aradului 119, 300645 Timisoara, Romania; magdalena.osiceanu@usvt.ro (M.O.); iuliana_popescu@usvt.ro (I.P.); alinaneacsu@usvt.ro (A.-G.N.); cosmin_popescu@usvt.ro (C.A.P.); georgeta_pop@usvt.ro (G.P.); simona_nita@usvt.ro (S.N.); florin_imbrea@usvt.ro (F.I.); dianaobistioiu@usvt.ro (D.O.); 3 Department of Biochemistry, Faculty of Life Science, Ahmadu Bello University, Zaria 810107, Kaduna State, Nigeria; masuleiman@abu.edu.ng; 4 Faculty of Veterinary Medicine, University of Life Sciences “King Michael I” from Timisoara, Calea Aradului 119, 300645 Timisoara, Romania; calin.hulea@usvt.ro

**Keywords:** Romanian *Hyssopus* essential oil, GC-MS, inhibition of haemolysis, protein denaturation, antimicrobial activity, molecular docking

## Abstract

This study compares three Romanian *Hyssopus officinalis* species—*H. officinalis* f. *ruber* (HOR), *H. officinalis* f. *albus* (HOA), and *H. officinalis* f. *cyaneus* (HOC)—evaluating their chemical composition and biological activities, specifically protein denaturation, haemolysis inhibition, and antibacterial effects. Chemical profiles were determined using Gas Chromatography–Mass Spectrometry (GC-MS). The species were cultivated at two distinct locations: the Didactic and Experimental Station DESUSVT and the Agricultural Research and Development Station Lovrin (ARDSL). This study investigates the correlation between chemical composition, biological activities, and local climate data at each site. The results show significant variations in chemical profiles, with species and cultivation location influencing the biological activities. *H. officinalis f. albus* (HOA) exhibited the strongest antimicrobial activity, particularly against Gram-positive bacteria. The molecular docking analysis highlighted key compounds, such as cyclohexene,4-isopropenyl-1-methoxymethoxymethyl and elemol, with binding solid affinities to microbial and inflammatory proteins. This study provides valuable insights into the chemical and biological properties of *Hyssopus officinalis*, emphasising its potential in combating microbial infections, protein denaturation, and haemolysis inhibition.

## 1. Introduction

Medicinal plants or herbs occupy a significant role in phytopharmaceuticals due to their varied biomolecules with biological activities. They demonstrate effectiveness against a range of pathologies, including diabetes, viral infections, bacterial diseases, and inflammatory conditions [1,2,3,4,5]. Having been used in traditional medicine since ancient times, these plants are now cultivated and rigorously tested to address the diminishing effectiveness of synthetic products in agriculture and health sectors.

*Hyssopus officinalis* is a polymorphous self-fertile medicinal plant species of the *Lamiaceae* family that grows as a perennial shrub or subshrub. Among the characteristic biological activities of *Hyssopus* spp. are mentioned as having curative properties against cough, loss of appetite, and spasmodic conditions; antibacterial and antimycotic activity; and insect-killing and antiviral properties. In addition to its biological activity, it is cultivated and used in the cosmetic and food industries for its aroma [6]. However, *Hyssopus officinalis* biological properties are different depending on the chemical composition of the plants, which is variable depending on the climate, the geographical area, the harvest period, the stages of development, and the part of the plant used. Moreover, extracting the bioactive compounds to obtain different product types represents another factor influencing biological activity [6]. *Hyssopus* essential oils are frequently extracted using traditional methods, such as Soxhlet extraction and hydrodistillation. Still, nonconventional methods, such as instant controlled pressure drop process and ultrasound-assisted and supercritical fluid extraction, have recently been used [6].

Although the essential oils of *Hyssopus officinalis* are chemically characterised by the predominance of monoterpene ketones, in particular pinocamphone or *cis*-pinocamphone, in variable concentrations [7,8,9,10,11,12,13,14,15,16], differences were observed in the literature depending on the region of the world. For example, the essential oil obtained from *H. officinalis* collected from the mountainous areas of Kurdistan is rich in camphor (23.61%) and β-pinene (21.91%) [17]. Instead, the one from Turkey contains mainly pinocarvone (29.2%) and *trans*-pine camphene (27.2%) [18], while the significant compounds in Spanish *H. officinalis* essential oil are represented by 1,8-cineole (53%) and β-pinene (16%) [19]. Nevertheless, most studies showed that the primary compounds are pinocamphone in *trans* (-) iso and *cis* forms [7,8,9,11,12,13,14,15,16]. However, the concentration of each form is variable depending on external (climate and geographical area) and internal factors (development, the age of the plant, and genetic factors). It was demonstrated that essential oils obtained from the plants grown below the 100 m above sea level contour contained high concentrations of pinocamphone. In comparison, the ones obtained from the 1000 m above sea level contour are characterised by the predominance of isopinocamphone [20]. Moreover, high concentrations of pinocamphone are observed before flowering and in the first year of plant growth, while isopinocamphone dominates during flowering and in three-year-old plants [21]. Regarding the influence of genetic factors, it was demonstrated that *H. officinalis f. albus* essential oil contains the highest concentrations of *cis*-pinocamphone (45.1%), followed by *H. officinalis f. cyaneus* (38.8%), while *H. officinalis f. ruber* essential oil was characterised by the predominance of *trans*-pinocamphone (58.3%) and only a percentage of 16.1% *cis*-pinocamphone [10]. By studying the diversity of the essential oils of *H. officinalis*, different genotypes from Moldova, Romania, Gonceariuc et al. [22] demonstrated that *cis* (-) pinocamphone (51.77%) was the dominant compound for *H. officinalis f. cyaneus*, *trans* (-) iso-pinocamphone (61.1%) for *Hyssopus officinalis f. albus*. At the same time, the concentration of each one was almost equal for *H. officinalis f. ruber*. As it is mentioned, many factors are responsible for the chemical composition. Still, the main compounds characteristic of this essential oil besides pinocamphone are α-pinene/β-pinene, sabinene, myrcene, phellandrene, linalool, myrtenol, elemol, and germacrene-D [23].

Of all the pharmacodynamic properties of *H. officinalis* essential oil, nowadays, particular importance is attributed to the antimicrobial effect. This statement is supported by finding alternative methods of combating and treating microbial and fungal diseases, which is essential since the emergence of multi-drug resistant (MDR) strains to most synthetic products is a global problem [24,25,26,27]. On the other hand, a natural antibacterial therapy should consider the bacteriostatic/bactericidal effect and the anti-inflammatory activity, knowing that bacterial LPS (lipopolysaccharide) activates monocytes and neutrophils [28,29,30]. Moreover, a recent study found that different bacterial pathogens, either alone or in combination, directly contribute to the pathogenesis of rheumatoid arthritis, a chronic inflammatory disorder [31]. The antimicrobial effect of *Hyssopus* essential oils was demonstrated against both Gram-positive and Gram-negative bacteria, such as *Staphylococcus aureus*, *S. epidermidis*, *Bacillus subtilis*, *Micrococcus luteus*, *Streptococcus pyogenes*, *S. pneumoniae*, *S. mutans*, *Listeria monocytogenes*, *Escherichia coli*, *Klebsiella pneumoniae*, *Salmonella* spp., *Pseudomonas aeruginosa*, and *Proteus mirabilis.* However, the MIC values differed depending on the type of bacteria tested, between 0.312–10 mg/mL for Gram-positive and 5–10 mg/mL for Gram-negative ones [16,17,32]. The difference in the cell envelope justifies the low or moderate antimicrobial activity against Gram-negative bacteria compared to Gram-positive ones. Being surrounded in addition by an outer membrane containing lipopolysaccharide, the Gram-negative bacteria tend to be more resistant to different natural or synthetic substances with antimicrobial activity [33].

Molecular docking has contributed significantly to understanding the possible molecular interactions between ligands (compounds) and microbial proteins, thus enabling the path to successfully identify and investigate good biological candidates with antimicrobial and anti-inflammatory potential [34,35]. Moreover, plants can produce antimicrobial and anti-inflammatory bioactive compounds of varying classes, ranging from flavonoids, phenolic acids, coumarins, tannins, quinones, stilbenes, and lignans. The antimicrobial and anti-inflammatory activity strongly depends on the protein’s amino acid residues, protein structure, and protein physicochemical properties [36]. DNA gyrase, a major bacterial protein involved in nucleic acid replication and transcription, has gained the attention of most antibiotic agents because of its high therapeutic index, wherein the enzyme catalysis of the negative supercoiling of bacterial DNA is altered by the inhibition of type II and IV topoisomerases [37]. The NF-κB is the regulator of proinflammatory cytokines in inflammation. Upon extended production, the activated and inactivated forms (p50–p65 heterodimer and p50 homodimer) of NF-κB would lead to systemic damage in uncontrolled inflammation [38,39]. This intricate property of the cytokine has rendered NF-κB a plausible anti-inflammatory target in prevailing over dysregulated inflammation [40]. Hence, the current study will use the in silico approach to show how the identified compounds from the plants can interact with the protein targets and exhibit putative antimicrobial and anti-inflammatory properties.

This study aims to compare and evaluate the chemical composition, determined by a CG-MS (Gas Chromatography–Mass Spectrometry) analysis, and the biological activities (such as antimicrobial activity, protein denaturation, and haemolysis inhibition) of three Romanian *Hyssopus officinalis* species. The seeds that were the basis for establishing the culture came from the Institute of Plant Genetics and Physiology of the Academy of Sciences of Moldova (Republic of Moldova). The seeds used in the present study were collected from a single population and sown in the two selected locations. The experimental design was selected to ensure no variation in the genotype so that the existence of different genes did not affect the comparison of the chemical composition and the biological activity. The selected species, *H. officinalis f. ruber* (HOR), *H. officinalis f. albus* (HOA), and *H. officinalis f. cyaneus* (HOC), were grown in two different research sites: Agricultural Research and Development Station Lovrin (ARDSL) (45.9684, 20.7726) and Didactic and Experimental Station DESUSVT (45.7855, 21.2098). The study also involves the correlation of the chemical composition with climate and environment data specific to the research locations and molecular docking that evaluates the connection between the plant’s chemical profile and related biological activities.

## 2. Results

### 2.1. Exploring the Effect of Soil and Climate Variability on Oil Yield Extraction

**Figure 1:**
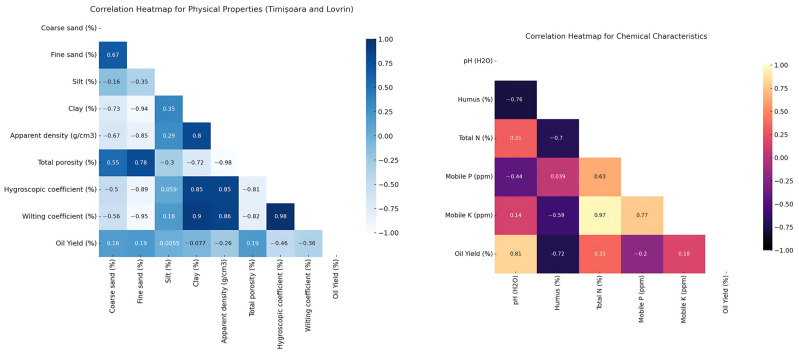
The Pearson correlation matrix for soil physical and chemical characteristics in Timișoara and Lovrin and the influence of oil yield extraction traits.

ANOVA results reveal significant differences in soil physical and chemical characteristics between the two locations studied (Table 1). Significant correlations also appear among structural variables when assessing soil physical properties (Figure 1).

The results indicate a weak positive relationship between coarse sand content and oil yield (+0.16). At the same time, total porosity is strongly negatively correlated with oil yield (−0.98), suggesting that highly porous soils are not necessarily favourable for oil production. Additionally, the wilting coefficient, which measures soil water retention under drought conditions, negatively correlates with oil yield (−0.36), suggesting that excessively dry soils limit productivity (Figure 1).

Considering the chemical properties, it is observed that pH values are closely linked to humus content, indicating an inverse relationship between soil acidity and the amount of organic matter present. Humus shows a negative correlation with oil yield (−0.72), which could suggest that an excessive accumulation of organic matter does not always favour increased production. This can be explained by the fact that too much humus may result in overly rich but less aerated soil, thereby reducing the efficiency of nutrient uptake essential for oil production. Furthermore, mobile potassium and phosphorus are strongly correlated (+0.97), indicating that these nutrients are often found in fertile soils and may contribute to a balanced nutrient supply. The Heatmap of Pearson correlation coefficients for chemical composition is available as Supplementary Materials Figure S1.

In contrast, the analysis of variance (ANOVA) for climatic conditions did not reveal significant differences in temperature and precipitation between the studied locations. The statistical values for temperature (F = 0.002, *p* = 0.96) and precipitation (F = 0.21, *p* = 0.64) indicate that in both the Lovrin and Timișoara regions, these climatic variables did not have a significant effect on the study results (Table 1). Thus, physical and chemical soil factors are more critical in influencing productivity.

### 2.2. Chemical Composition of HOEOs by GC-MS

The GC-MS analysis results are presented in Table 2.

The analyses show that although climatic influences are common between Timisoara and Lovrin, these are not the main factors influencing the chemical composition of the hyssop genotypes studied. The chemical composition is influenced by the type of variety used. The high correlations mainly reflect genetic and local environmental similarities rather than direct temperature and precipitation influences.

**Figure 2:**
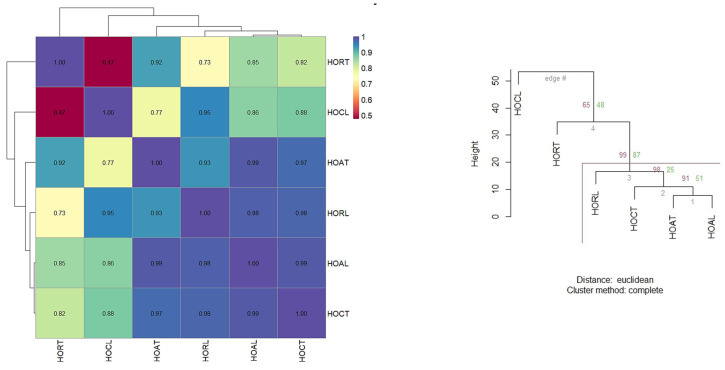
The heatmap of Pearson correlation coefficients for chemical composition by variety and location.

The results obtained indicate that HORT and HORL have a strong correlation (0.73), HOAT and HOAL have an extremely high correlation (0.99), and HOCT and HOCL have a robust correlation (0.97) (Figure 2). Thus, the chemical composition of essential oils is strongly determined by the variety’s genetics, which explains the high similarities.

Although climatic and soil influences are present and could have an effect, they do not seem to significantly alter the chemical composition of essential oils to the extent that genetics, with respect to the type of variety, do. The local microclimate and soil characteristics may introduce minor variations, but they do not change the basis of the chemical composition of the varieties.

The most significant differences in chemical composition are observed between HORT and HOCL, reflecting the importance and impact of genetic factors in different growing conditions.

The HOR variety showed the most considerable variations depending on the locations used, indicating sensitivity to local environmental and soil conditions, requiring customised agricultural practices to ensure the consistency of the chemical composition. On the other hand, the high similarity between locations observed in the HOA and HOC varieties indicates that these varieties are very stable and suitable for various locations without compromising the quality of the essential oils (Figure 2).

**Figure 3:**
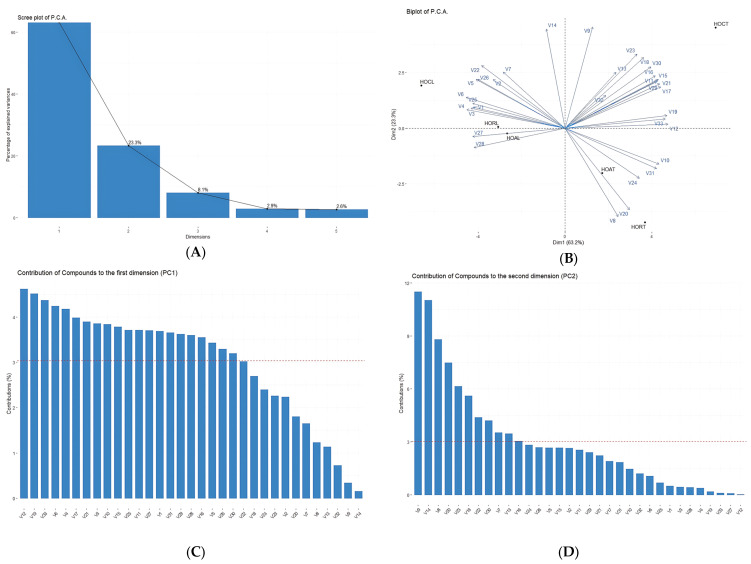
(**A**) The scree plot of P.C.A.; (**B**) the biplot of P.C.A.; (**C**) the contribution of variables to the first dimension of P.C.A.; (**D**) the contribution of variables to the second dimension of P.C.A. Note: V1: α-Pinene, V2: α-Phellandrene, V3: β-Pinene, V4: Sabinene, V5: β-Myrcene, V6: Limonene, V7: β-Phellandrene, V8: *β-cis*-Ocimene, V9: Eucalyptol, V10: cyclohexene, 4-isopropenyl-1-methoxymethoxymethyl-, V11: α-Gurjunene, V12: β-Bourbonene, V13: linalool, V14: 3-Thujanone, V15: Caryophyllene, V16: Alloaromadendrene, V17: Humulene, V18: α-Caryophyllene, V19: γ-Cadinene, V20: Pinocamphone, V21: germacrene d, V22: Isocamphopinone, V23: γ-Elemene, V24: Estragole, V25: 3-octen-5-yne, 2,7-dimethyl-, (e)-, V26: p-menth-1-en-8-ol, V27: (1r)-(−)-Myrtenal, V28: myrtenol, V29: Ledol, V30: elemol, V31: Eugenol methyl ether, V32: Caryophyllene oxide, V33: (−)-Spathulenol.

Following the PCA analyses presented in Figure 3, pinocamphone (**20**) and isopinocamphone (**21**) are distinctive compounds for the HORT and HOCL varieties studied. These compounds are extreme values, indicating significant differences in the chemical composition of these varieties. Also, most of the compounds analysed are grouped close to the origin, suggesting that the chemical profiles of these compounds are similar between most of the varieties studied and for the two locations studied, suggesting an insignificant influence of the environment regarding the chemical components. Varieties of chemical components such as HOAT, HOAL, HOCT, and HORL indicate less variation in chemical components. Another component that shows variation is β-pinene (**3**), which is not centrally located and indicates a significant variation compared to the other compounds, being more distinct.

### 2.3. Inhibition of Haemolysis Values and Protein Denaturation

#### Inhibition of Haemolysis Values

The results of the inhibition of haemolysis (IH%), inhibition of protein denaturation (IPD%), and IC50 values of different HOEOS are presented in Table 3. Samples HOAL and HOCT demonstrate the ability to protect against haemolysis, starting at 1 mg/mL, with an inhibition percentage value of 13.17% and 16.09%, respectively. Sample HOAT shows the ability to inhibit haemolysis starting with the concentration of 8 mg/mL in a proportion of 16.12%, the values of the percentage of inhibition following an upward trend as the oil concentration increases. In the case of samples HORT and HORL, the percentage of inhibition has a positive value from the concentration of 8 mg/mL, protection against haemolysis being higher for sample HORT (5.01%) compared to sample HORL (0.61%). In the case of HOCL, the protection of the lysosomal membrane is demonstrated only at a concentration of 32 mg/mL in a proportion of 13.23%.

### 2.4. The Antimicrobial Activity of HOEOs

Table 4 presents the bacterial inhibition rate (BIR%) values of *Hyssopus officinalis* essential oils (HOEOs) obtained according to Formula (4) given in 4.6. The BIR% values are expressed as percentages reported to the positive controls.

The antimicrobial research presents a picture that promotes efficacy against Gram-positive bacteria compared to Gram-negative bacteria. The Gram-positive bacteria were inhibited more by HOA, *B. cereus*, and *C. perfringens*, which were positive bacteria that were inhibited more by HOA, *B. cereus*, and *C. perfringens*, being the only two strains that required a higher concentration to reach the MIC. Concerning the efficacy analysis, the results are as follows HOA > HOC > HOR (>—more efficient). Regarding the Gram-negative bacteria, HOA and HOC proved to have antibacterial efficacy, and HOA and HOC proved antibacterial efficacy only starting at 8 mg/mL. Analysing the antibacterial activity, *C. perfringens* and *P. aeruginosa* proved to be the most resistant strains to the activity of HOEOs, the inhibitory rate being negatively correlated to the increase in concentration, negative rate sustained by a decreasing efficacy expressed as BIR% that reached negative values alongside the increase in concentration.

Summarising the HOEOs’ activity against Gram-positive and Gram-negative bacteria, HOAL was the most effective, showing the highest inhibition rates against *S. pyogenes*, *S. aureus*, *B. cereus*, *C. perfringens*, *S. flexneri*, *E. coli*, and *H. influenzae*. HOAT proved the most effective against *P. aeruginosa*, while HOCL reached the highest BIR% on the *S. typhimurium* strain. Regarding the MIC values, HOA showed the lowest MIC, followed by HOC and HOR. Concerning the MIC values, HOA showed the lowest MIC, followed by HOC and HOR, which demonstrated a MIC mostly at 8 mg/mL.

### 2.5. Molecular Docking

**Figure 4:**
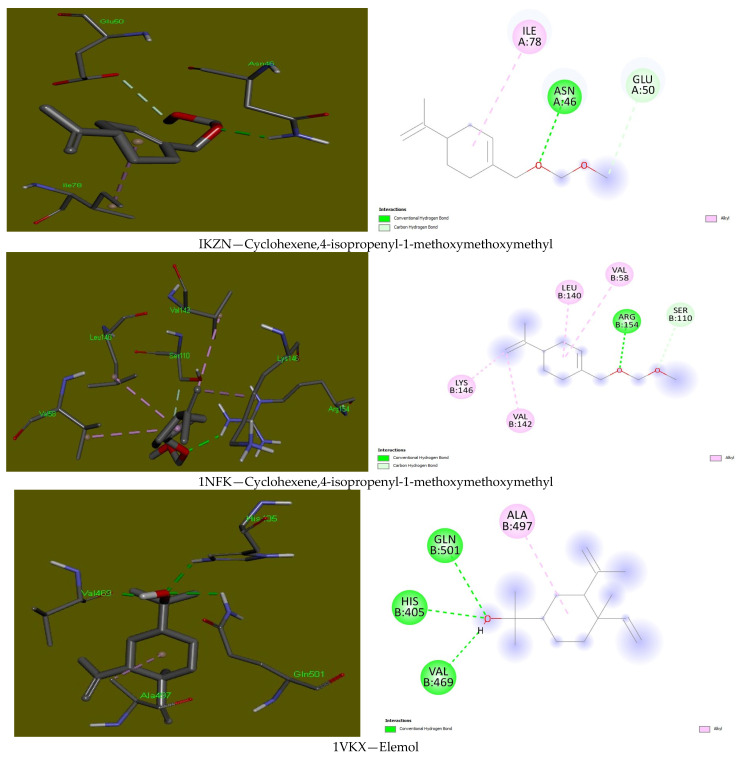
Three-dimensional (3D) and two-dimensional (2D) structural best binding modes between the antimicrobial, anti-inflammatory proteins and the identified compounds of the Hyssopus plants.

The molecular docking results against DNA gyrase protein (1KZN) showed cyclohexene,4-isopropenyl-1-methoxymethoxymethyl having the best binding affinity for this protein with a binding energy of −5.8 kcal/mol (Figure 4). In a similar binding interaction, cyclohexene,4-isopropenyl-1-methoxymethoxymethyl also interacted with the inflammatory protein, p50-homodimer (1NFK-protein complex, which plays a critical role in regulating the immune response to infection, inflammation, and stress), having a docking score of −4.9 kcal/mol. However, elemol (−5.9 kcal/mol) gave the best binding interaction with the p50–p65 heterodimer protein of the inflammatory cytokine (1KVX). Interestingly, all the interactions revealed a stable complex formation between these compounds and the amino acid residues owing to the strength of the affinity displayed amongst other tightly bonded hydrophobic interactions formed with other docked compounds (Table 5).

In the binding mode of 1KZN, the two-oxygen group of cyclohexene,4-isopropenyl-1-methoxymethoxymethyl formed the hydrogen bond with Asn46 and the carbon–hydrogen bond with Glu50, while the benzene group formed an alkyl hydrophobic interaction with Ile78. Similarly, this compound interacted with 1NFK in the same fashion with the carbonyl group (H: Arg154, C-H: Ser110) of the compound and four hydrophobic bonds involving Val58, Leu140, Val142, Lys146. On the contrary, elemol interacted best with 1VKX, His405, Val469, and Gln501, which were involved in the hydrogen bonds. At the same time, Ala497 formed the alkyl hydrophobic interaction, thus suggesting a high affinity of the compound to the protein and could be responsible for the inhibitory mechanism of action of the compound (Figure 4).

## 3. Discussion

### 3.1. Climate Factor Data

Romania is a Carpatho-Danubian-Pontic country that has a transitional temperate continental climate. Across the 238,391 km^2^ of Romanian territory, there are distinguished oceanic influences in the western and central regions, sub-Mediterranean climate in the southwest, continental-excessive climate in the southeast and east, Scandinavian–Baltic climate in the northeast of the country, Pontic climate in the southeast, and transitional climatic influences in the southern part [41,42]. Like other countries worldwide, climate changes have been noticed in Romanian territory over the last decades, such as heat waves [43,44] and drought [45]. Moreover, on a national level, the studies and the observation data in the last 40 years revealed an increase in temperature in almost all months of the year. This changed precipitation regime decreased in the first six months of the year and registered a slight increase in the other six [41]. Besides the fact that the determination of annual temperature and precipitation is an essential tool in identifying climate changes, correlations of climatic aspects with the chemical composition of some cultivated medicinal plants allow for the identification of the plant’s needs to obtain, in the future, plants with complex biological activity through the phyto-substances contained.

The *H. officials* plant taken into the study was cultivated in western Romania, a region characterised by higher humidity, abundant precipitation, and westerly winds. According to the present study, the average annual temperature in the two studied areas of western Romania was around 12 °C, with 45–50 mm of precipitation. No difference was observed regarding the seasonal temperature between the studied regions. However, in the summer and autumn seasons, the average rainfall was higher in Lovrin (57 mm and 47 mm) than in Timișoara (43 mm and 36 mm) but lower in the winter.

### 3.2. Gas Chromatography–Mass Spectrometry (GC/MS)

Various soil–climatic and genetic factors influence the appearance of different chemotypes (pinocamphonic, linalool, thymol) of the essential oil of *Hyssopus officinalis* L. [46,47]. The same factors influence the quantity of various compounds. Nevertheless, the main components characteristic of the plant are pinocamphone and isopinocamphone, β pinene, sabinene, myrcene, linalool, myrtenol, elemol, and germacrene-D in different concentrations [7,8,10,11,12,13,14,15,16].

As the literature described from all the chemotypes, the pinocamphone one seems to be frequently identified in different regions of the world, with variable concentrations of the main compounds [7,8,11,12,13,14,15,16]. The present study demonstrated that the *Hyssopus officinalis* essential oils contained pinocamphone in concentrations between 6.09 and 45.96% and isopinocamphone between 20.09 and 54.75% as main compounds. Other compounds consisted of β-pinene (8.74–15.84%), germacrene D (2.76–5.77%), γ-elemene (2.22–3.82%), and caryophyllene (1.00–5.12%). Instead, Said-Al Ahl et al. [48] sustained that essential *H. officinalis* oil from Egypt contains *cis*-pinocamphone as a majority compound in a concentration of 26.85%, followed by β-pinene (20.43%) and *trans*-pinocamphone (15.97%). The one from Iran contains a concentration of *cis*-pinocamphone of 21.59%, followed by 7.93% of *trans*-pinocamphone and 7.12% of elemol [49]. Oils from *Hyssopus officinalis* found in Poland were chemically characterised by the predominance of isopinocamphone in concentrations between 22.53 and 28.74%, followed by pinocamphone (11.41–17.99%) and β-pinene (6.69–12.01%) [14]. The same main compounds were identified in the Spanish oils in various concentrations: isopinocamphone—24.6%, pinocamphone—19.8%, and β-pinene—≤20.5% [50,51].

The concentration of each compound varied between and within the same variety in the two regions taken into study. Except for HORT, isopinocamphone was the primary compound, HOCT being representative of the highest concentration (54.75%), while the lowest was detected in HOCT (33.97%). Pinocamphone was the primary compound observed in the case of HORT, while the lowest concentration was observed in the case of HOCT. Except for HOCT, the third primary compound, β-pinene, was around the concentration of 10%. However, except for HOA, there was an observed difference between the essential oils from the two regions studied regarding the first three chemical compounds. HORT was characterised by higher concentrations of pinocamphone and a concentration of almost half of isopinocamphone, while in the case of HORL, the major compounds were reversed. In the case of HOCT and HOCL, the main compounds were also the two bicyclic monoterpenes ketones, mentioning that for HOCL (6.09%), the concentration of pinocamphone was almost half that of HOCT (22.17%).

Moreover, HOCT was characterised by almost double concentrations of β-pinene, 15.85%, compared to HOCT. These differences in the chemical composition of identical varieties demonstrate that additional factors, such as nutrient levels, the soil type, other climatic variables (light, carbon dioxide, and moisture), and the growth stage, can also influence total phytochemical content [52,53]. Moreover, these factors seem decisive since the flowers’ colour has no significant effect on the amount of essential oil, as the literature sustained [54].

### 3.3. Inhibition of Haemolysis and Protein Denaturation

According to the literature, natural products’ capacity to ensure erythrocyte membrane stabilisation is associated with anti-inflammatory activity [5,55,56]. Since the human red blood cell membrane is analogous to the lysosomal membrane, the integrity of the last one is vital in controlling the inflammatory response by inhibiting the release of proteases and bactericidal enzymes of the activated neutrophils [57]. Moreover, protein denaturation by losing their quaternary, tertiary, and secondary structures, associated with losing their biological functions, may be a marker for chronic inflammatory and arthritic diseases [5,55,56]. Therefore, the development of new natural anti-inflammatory medications should consider these two aspects: inhibiting the release of proteases of activated neutrophils and preventing the loss of protein structure.

This study demonstrates that HOEO exhibits anti-inflammatory activity, with IC_50_ values for haemolysis inhibition ranging from 3.82 to 7.17 mg/mL and for protein denaturation from 12.99 to 24.36 mg/mL. Differences regarding these properties were observed between each type of HOEO, the most efficient being HOCT. The results contradict Micovic et al. [32], which states that essential oils exhibited no significant activity, while *H. officinalis* extracts at a concentration of 20 μg/mL showed a percentage of the inhibition of COX-2 enzyme almost similar to celecoxib at a concentration of 8.8 μM. Moreover, a concentration of 200 mg/kg extract showed an inhibitory effect on the increase in rat paw oedema in the third and fourth hours after carrageenan administration [32]. On the other hand, it was demonstrated that *H. officinalis* extract inhibited the invasion of eosinophils and affected interleukin-4, -6, and -17 and interferon-γ levels in asthmatic mice [38].

From all the compounds, it was demonstrated that pinene, similar to α-pinene, had anti-inflammatory activities by decreasing IL-4 and IL-13 gene expression in LPS-stimulated RBL-2H3 cells [58]. Other compounds known for their antioxidant, anti-inflammatory, and re-epithelialisation activities are terpinolene, α-phellandrene, and β-caryophyllene [25,59]. However, the synergism or antagonism activity of these compounds and others in essential oils justifies various responses.

The differences observed regarding anti-inflammatory activity between our study and the others from the literature can be sustained by different extraction methods and, respectively, different chemical compositions. On the other hand, various methods were used to test this biological property. However, other in vivo and in vitro tests are recommended to complete the results obtained in the present study.

### 3.4. The Antimicrobial Activity of HOEOs

Like other biological activities, the antimicrobial one of HOEO depends on the chemical composition, which is influenced by internal or external factors, and the bacterial strain tested. This explains the different results regarding the minimum inhibitory concentration obtained in the studies researchers conducted worldwide [11,16,60,61]. Micovic et al. [60] demonstrated that HOEO from Montenegro and Serbia had a MIC value of 400 µg/mL against *S. aureus*, while Baj et al. [16] showed that the one from Poland had a higher value of MIC against the same strain, respectively, between 5 mg/mL and 10 mg/mL. Similarly, Stappen et al. [61] found that high concentrations of HOEO Himalaya, 4000 µg/mL, are necessary to inhibit the growth of *S. aureus*. Instead, the results of the present study showed a value of MIC of 1 mg/mL not only against *S. aureus* but also against *L. monocytogenes* and *S. pyogenes*, even if Moloudi et al. [17] claimed a decreased MIC value against *L. monocytogenes*, 312 µg/mL. No difference was observed regarding the antimicrobial activity against these strains of the studied essential oils. However, the MIC values against *B. cereus* varied between 1 and 8 mg/mL, the most efficient against this strain being HOAT. In contrast, Acimovic et al. [11] found that the growth of the *B. cereus* strain was inhibited at a concentration of HOEO of 14.20 µL/mL. All the essential oils studied, HORL, HORT, and HOCL, demonstrated the lowest antibacterial activity against this Gram-positive bacteria. The anaerobe, Gram-positive bacteria *C. perfringens*, seems resistant to the HOEO, except for HOAT, which had a MIC value of 1 mg/mL.

Also, the MIC values against Gram-negative bacteria were variable and generally higher due to the different structures of the cell walls of the two groups of bacteria. Thus, the MIC values of the studied HOEO were between 1 and 2 mg/mL against *S. typhimurium*, 1 and 4 mg/mL against *P. aeruginosa*, and 1 and 8 mg/mL against *E.coli*, *S. flexneri*, and *H. influenzae*. Similarly, Stappen et al. [61] stated that concentrations of 2000 µg/mL and 8000 µg/mL are considered MIC values for *E. coli* and *P. aeruginosa*, while Baj et al. [16] demonstrated that a 5 mg/mL concentration is efficient against both bacteria. In contrast, Acimovic et al. [11] showed that MIC values were 227.25 µL/mL against *E. coli* and *S. enteritidis* and 454.50 µL/mL against *P. aeruginosa*.

Even though the studied HOEO seems to have the same antimicrobial properties as others from various world regions [16,32,61], differences were observed between and within the same genotype in the two studied areas. HOAT was generally the most efficient against Gram-negative bacteria, followed by HOC and HOR, regardless of location.

The antimicrobial activity of HOEO is mainly due to monoterpene ketones. Also, alcoholic monoterpenes, such as linalool, are recognised as bactericidal agents [62]. β-pinene, an isomer of pinene with an exocyclic double bond, along with α-pinene, are described in the literature as a “miracle gift of nature” due to their therapeutic potential, including antimicrobial activity [63]. From the sesquiterpene group, caryophyllene and germacrene-D present strong antibacterial effects [3,64,65]. Since all these represent the main compounds of the tested oil, their antibacterial efficiency is justified. However, the lower antimicrobial activity of some varieties compared to others is due to the antagonistic activity of some chemical compounds found in lower concentrations.

### 3.5. Molecular Docking

The docking results from this study have revealed some fascinating interactions between the compounds of the plants and the amino acids of the three protein targets, respectively. This is owing to the formed hydrogen bonds with the oxygen and hydroxyl side groups and the many tightly bonded hydrophobic interactions with the benzyl ring and its side groups of the other compounds. Cyclohexene,4-isopropenyl-1-methoxymethoxymethyl and elemol are the two compounds responsible for the high binding affinity with the proteins. The study established a docked region with DNA gyrase that corresponds with the research by Dharani et al. [66], wherein amino acid residues in the binding region had a similar bonding pattern to our study. As for the interaction with proinflammatory cytokines, the docking underscores two possible high-binding complexes with the two forms of the proteins. The binding sites of cyclohexene,4-isopropenyl-1-methoxymethoxymethyl and elemol suggest the anti-inflammatory potential of these two compounds in comparison with the others. These compounds shared a binding site closest to the proteins’ DNA binding region. Furthermore, the nature of their binding interaction will infer to the extent of stability the binding complex complements with the affinity of the compounds to the proteins and, therefore, affects the binding of the proteins to DNA [67,68]. Ultimately, the deduced inhibitory prediction of these compounds from Hyssopus plants is worthy of being subjected to more antimicrobial and anti-inflammatory assays to ascertain their efficacy as promising biological candidates.

## 4. Materials and Methods

### 4.1. Chemicals

All reagents used for chemical analysis were purchased from Sigma–Aldrich Chemie GmbH (München, Germany) and Geyer GmbH (Renningen, Germany) and were of analytical quality.

### 4.2. Samples

The three Romanian *Hyssopus officinalis* types taken into study were *H. officinalis f. ruber*, *H. officinalis f. albus*, and *H. officinalis f. cyaneus*. The plants were cultivated at two distinct locations: the Agricultural Research and Development Station Lovrin (ARDSL) and the Didactic and Experimental Station DESUSVT, with coordinates (45.9684, 20.7726) and (45.7855, 21.2098), respectively. The plants were harvested during the optimal vegetation period when more than 50% of the inflorescences bloomed.

To ensure the experimental design, voucher specimens were botanically identified and deposited in a temperature-controlled herbarium (22–25 °C and 30–40% relative humidity) in the Botany Department at the University of Life Sciences King Michael I of Romania in Timişoara, with the codes: *H. officinalis* subsp. *officinalis* var. *vulgaris f. cyaneus* from DESUSVT (HOCT) VSNH.ULST-BD96, *H. officinalis* subsp. *officinalis* var. *vulgaris f. cyaneus* from ARDSL (HOCL) VSNH.ULST-BD97, *H. officinalis* subsp. *officinalis* var. *vulgaris f. ruber* from DESUSVT (HORT) VSNH.ULST-BD98, *H. officinalis* subsp. *officinalis* var. *vulgaris f. ruber* from ARDSL (HORL) VSNH.ULST-BD99, *H. officinalis* subsp. *officinalis* var. *vulgaris f. albus* from DESUSVT (HOAT) VSNH.ULST-BD100, and *H. officinalis* subsp. *officinalis* var. *vulgaris f. albus* from ARDSL (HOAL) VSNH.ULST-BD101.

The plants collected were dried at 40 °C and used for the essential oil extraction. The extraction was carried out from the herbal dry mass of the plant, respectively, the stem portions that include the inflorescences and leaves without the lignified basal part. The essential oil was extracted using hydrodistillation Clevenger equipment (Clevenger extractor, experimental model, Timisoara, Romania) for 4 h. The resulting essential oils and aromatic water mixture were separated using a separating funnel. The pure essential oils were stored in glass vials at +4 °C until further analysis. The yield of essential oil was determined by the gravimetric method and calculated in percentage of plant dry weight: HOAT 0.41%; HORT 0.72%; HOCT 0.81%; HOAL 0.56%; HORL 0.86%, and HOCL 0.83%.

### 4.3. Climate Data Evaluation

The data sets used for the meteorological analysis were collected from the Regional Meteorological Center in Banat-Crisana, Romania, and the Meteorological Station in Lovrin.

### 4.4. Gas Chromatography–Mass Spectrometry (GC/MS)

EO samples were chemically characterised using GC-MS. Analysis was conducted with a Shimadzu QP 2010 Plus apparatus (Columbia, SC, USA) equipped with an AT WAX capillary column (30 m, 0.32 mm, 1 µm) following the method presented in previous studies [69,70]. Helium was used as the carrier gas at a 1 mL/min flow rate, with the injector and ion source temperatures set at 250 °C and 220 °C, respectively. A temperature gradient was used for compound separation, starting with an initial oven temperature of 40 °C (held for 1 min), then a gradual increase to 210 °C at a rate of 5 °C/min, and a 5 min hold. The sample injection volume was 1 μL of a 2% EO/hexane solution, with a 1:50 split ratio.

NIST 5 Wiley 275 library was used to identify the volatile compounds, with a minimum identification accuracy of 90%. The results were presented as a proportion of the total compounds; quantification was conducted using the area normalisation method applied to SIM MS data acquisition. The Linear Retention Index (LRI) was calculated using a Normal alkane RI for the same polar column, with the values corresponding to the percentage area of the peaks for each compound identified [71].

### 4.5. Inhibition of Haemolysis Values and Protein Denaturation

#### 4.5.1. Membrane Lysis Assay

##### Preparation of Red Cell Suspension

The procedure outlined by Gunathilake et al. [55] was used to prepare the erythrocyte suspension with slight modifications. Human heparinised blood was centrifuged at 3000× *g* rpm for 10 min. After centrifugation, the supernatant was removed, and the erythrocyte mass was washed with an equal volume of isotonic sodium chloride solution (0.9%). The centrifugation and washing steps were repeated three times. Subsequently, the blood volume was measured and reconstituted as 40% suspension with isotonic PBS solution at a pH of 7.4. The erythrocyte suspension was prepared following the method described [55], with slight modifications [5,72]. Human heparinised blood was centrifuged at 3000× *g* rpm for 10 min. After centrifugation, the supernatant was discarded, and the erythrocyte pellet was washed with an equal volume of 0.9% isotonic sodium chloride solution. This centrifugation and washing process was repeated three times. Finally, the blood volume was measured and reconstituted as a 40% suspension in isotonic PBS solution with a pH of 7.4.

#### 4.5.2. Heat-Induced Haemolysis

The heat-induced haemolysis assay was performed following the method developed by Okoli et al. [73], with modifications [55]. Briefly, varying concentrations of essential oil (1 mg/mL, 2 mg/mL, 4 mg/mL, 8 mg/mL, 16 mg/mL, 32 mg/mL, and 64 mg/mL) were suspended in 5 mL of isotonic PBS solution (RemedLab, Bucharest, Romania) at pH 7.4. To each mixture, 100 µL of red blood cell suspension was added. After gentle shaking, the samples were incubated in a water bath at 54 °C for 20 min. Following incubation, the samples were centrifuged at 2500× *g* rpm for 3 min, and the absorbance of the supernatant was measured at 540 nm using a UV-VIS spectrophotometer (Specord 205; Analytik Jena AG, Jena, Germany). A negative control consisted of PBS and 100 µL of erythrocyte suspension, while the positive control was 0.1 mg/mL dexamethasone in 5 mL PBS and 100 µL erythrocyte suspension.

Formula (1) was used to determine the percentage of haemolysis inhibition:

(1) $$\%\;inhibition\;of\;haemolysis=100-\frac{A1}{A2}*100$$

where

*A*1 represents the absorbance of the tested sample;

*A*2 represents the absorbance of the negative control.

#### 4.5.3. The Effect on Protein Denaturation

The protein denaturation assay was carried out following the method described by Gunathilake et al. [55], with slight modifications. Different concentrations of the tested essential oil (1 mg/mL, 2 mg/mL, 4 mg/mL, 8 mg/mL, 16 mg/mL, 32 mg/mL, and 64 mg/mL) were added to 1 mL of 1% egg albumin (Oxford Lab Fine Chem, Maharashtra, India) and 4 mL of PBS (pH 6.4) (RemedLab, Bucharest, Romania). The mixture was incubated at 37 °C for 15 min and then heated to 70 °C for 5 min in a water bath (D-91126, Memmert GmbH & Co. KG, Schwabach, Germany). After cooling, the absorbance was measured at 660 nm using a UV-VIS spectrophotometer (Specord 205; Analytik Jena AG, Jena, Germany). The control solution consisted of albumin and PBS without essential oil.

The percentage inhibition of protein denaturation was calculated using Formula (2):

(2) $$\%\;inhibition=100-\frac{A1}{A2}*100$$

where

*A*1 represents the absorbance of the tested sample;

*A*2 represents the absorbance of the control.

### 4.6. Evaluation of the Antimicrobial Activity

The antimicrobial activity of HOEOs used in this study was evaluated through broth microdilution against various ATCC strains, categorised into Gram-positive and Gram-negative groups. The Gram-positive ATTC strains tested were *Streptococcus pyogenes* (ATCC 19615), *Staphylococcus aureus* (ATCC 25923), *C. perfringens* (ATCC 13124), *Listeria monocytogenes* (ATCC 19114), and *Bacillus cereus* (ATCC 10876). The Gram-negative strains taken into the study were *Pseudomonas aeruginosa* (ATCC 27853), *Shigella flexneri* (ATCC 12022), *Escherichia coli* (ATCC 25922), *Salmonella typhimurium* (ATCC 14028), and *Haemophilus influenzae* tip B (ATCC 10211).

All the bacterial strains are part of the culture collection maintained by the Laboratory of Microbiology at the Interdisciplinary Research Platform of the University of Life Sciences “King Mihai I of Romania” in Timișoara.

Our previous studies describe the method [53,74]. Briefly, a stock solution of essential oil was prepared in dimethylsulphoxide (DMSO), and then, 50 µL of stock solution containing different lavender essential oils tested were added over 100 µL freshly grown bacterial suspension diluted at an optical density (OD) of 0.5 McFarland standard (1.5 × 10^8^ UFC × mL). Serial dilutions were performed.

All six essential oils were tested at concentrations of 1 mg/mL, 2 mg/mL, 4 mg/mL, 8 mg/mL, 16 mg/mL, 32 mg/mL, and 64 mg/mL.

A pure uninhibited strain in BHI was used as a positive control.

The MIC, the lowest compound concentration that yields no visible microorganism growth, was determined by the measurement of OD using the spectrophotometric method [75,76].

Two indicators were calculated for interpreting the results—BGR (bacterial growth rate) and BIR (bacterial inhibition rate)—according to Formulas (3) and (4):

BGR = OD_sample_/OD_negative control_ × 100 (%) (3)

BIR = 100 − BGR (%)(4)

where OD_sample_ represents the optical density of the sample, containing a certain type and concentration of essential oils tested, at 540 nm as a mean value of triplicate readings; OD_negative control_ is the optical density at 540 nm as a mean value of triplicate readings for the selected bacteria in BHI.

### 4.7. Correlation Analysis and Path Analysis

The statistical analyses were performed using the R Studio v.4.1.1 software. Pearson correlations were used to assess the relationships between climatic variables (temperature and precipitation) and the chemical composition of essential oils. Principal component analysis (PCA) was used to reduce the dimensionality of the data and identify the most distinctive chemical compounds for each variety.

### 4.8. Molecular Docking Studies

The in silico study involved fourteen compounds (14) that were abundant in the Hyssopus plants and three (3) putative protein targets (PDB: 1KZN, 1NFK, 1VKX). The protein crystal structures were retrieved from https://www.rcsb.org (accessed on 13 July 2024) [77] and prepared for docking in UCSF chimera 1.17.3 by removing ligands, DNA, solvents, ions, and heteroatoms. The compounds were downloaded in SDF format from https://pubchem.ncbi.nlm.nih.gov (accessed on 13 July 2024) [78]. Molecular docking was carried out in the PyRx virtual screening program (Python Prescription 0.8). The grid boxes were set at maximum to cover all the proteins (macromolecules). The three-dimensional (3D) and two-dimensional (2D) interactions were visualised with the help of Discovery Studio Visualizer v21.1.0.20298 (BIOVIA, San Diego, CA, USA).

## 5. Conclusions

In our study, there were notable differences in the chemical profiles of *Hyssopus officinalis* essential oils (HOEOs) between the different species and growing locations. The two main chemicals were isopinocamphone and pinocamphone, and their quantities varied depending on genetic and environmental circumstances. These results emphasise the significance of location and genotype in influencing the composition of essential oils. *H. officinalis f. albus* proved the most effective antibacterial, especially against Gram-positive bacteria. The findings provide validity to HOEOs’ potential as synthetic antibacterial agent substitutes, mainly because of the rising incidence of antibiotic resistance. Additionally, the study used assays for haemolysis inhibition and protein denaturation to show that HOEOs have anti-inflammatory qualities. This study successfully employed molecular docking tools to establish the possible binding interactions between the compounds with high abundance and the microbial (1KZN) and inflammatory (1NFK, 1VKX) proteins. Cyclohexene,4-isopropenyl-1-methoxymethoxymethyl and elemol showed interesting binding affinity to the proteins and posed hydrogen and hydrophobic interactions in a drug-like fashion. Hopefully, these compounds will help us understand further how these plants retain their antimicrobial and anti-inflammatory properties.

The study concludes that the main chemical composition variables are genetics, with minor variances influenced by local environmental factors. The consistency of the chemical profiles across various sites highlights the resilience of several *Hyssopus officinalis* variants for growing in various conditions.

## Figures and Tables

**Table 1:** The analysis of variance for soil physical characteristics of the analysed locations.

Source of Variance	DF	SS (Sum of Squares)	F-Statistic	*p*-Value	Significance
Coarse sand (%)	1	6.72	34.21	3.829 × 10^−4^	*p* < 0.001 ***
Fine sand (%)	1	146.69	23.48	1.279 × 10^−3^	*p* < 0.01 **
Silt (%)	1	0.49	0.13	7.200 × 10^−1^	ns
Clay (%)	1	178.08	24.99	1.054 × 10^−3^	*p* < 0.01 **
Apparent density (g/cm^3^)	1	0.19	27.78	7.545 × 10^−4^	*p* < 0.001 ***
Total porosity (%)	1	184.9	12.62	7.481 × 10^−3^	*p* < 0.01 **
Hygroscopic coefficient (%)	1	2.73	13.85	5.856 × 10^−3^	*p* < 0.01 **
Wilting coefficient (%)	1	8.63	17.33	3.151 × 10^−3^	*p* < 0.01 **
pH in H_2_O	1	6.72	1.08	3.27 × 10^−1^	ns
Humus (%)	1	146.69	7.04	4.52 × 10^−2^	*p* < 0.05 *
N total (%)	1	0.49	284.53	1.34 × 10^−5^	*p* < 0.001 ***
P mobil (ppm)	1	178.08	2.58	1.69 × 10^−1^	ns
K mobil (ppm)	1	184.9	60.65	5.59 × 10^−4^	*p* < 0.001 ***
Temperature	1	0.15	0.002	0.96	ns
Precipitation	1	140.16	0.21	0.64	ns

*** *p* < 0.001, ** *p* < 0.01, * *p* < 0.05, ns: non-significant.

**Table 2:** The chemical composition of the HOEOs analysed by GC-MS.

No. crt	RI c/ Rir	Compounds	HORT (%)	HORL (%)	HOAT (%)	HOAL (%)	HOCT (%)	HOCL (%)
1	1021/1015	α-Pinene	0.49	0.60	0.49	0.54	0.44	0.82
2	1048/1050	Camphene	0.19	0.22	0.18	0.19	0.20	0.26
3	1104/1096	β-Pinene	9.45	11.77	10.37	10.86	8.74	15.85
4	1136/1140	Sabinene	1.44	1.84	1.40	1.65	1.30	2.20
5	1158/1164	β-Myrcene	1.28	2.02	1.45	1.92	1.55	2.05
6	1196/1193	Limonene	0.66	0.93	0.77	0.94	0.71	1.00
7	1211/1209	β-Phellandrene	1.20	4.31	2.15	4.16	2.94	3.28
8	1232/1234	β-*cis*-Ocimene	0.98	0.19	0.80	0.28	0.24	0.11
9	1240/1237	Eucalyptol	0.14	0.19	0.15	0.19	0.27	0.19
10	1494/1490	Cyclohexene, 4-isopropenyl-1-methoxymethoxymethyl-	2.17	1.47	1.64	1.55	1.94	1.14
11	1528/1528	α-Gurjunene	0.38	0.29	0.41	0.27	0.64	0.28
12	1532/1533	β- Bourbonene	1.75	0.63	1.41	0.75	2.16	0.53
13	1545/1542	Linalool	0.68	0.67	0.79	0.88	0.93	0.69
14	1570/1568	3-Thujanone	0.00	0.07	0.00	0.03	0.10	0.09
15	1585/1588	β-Caryophyllene	2.37	1.25	2.12	1.31	5.12	1.01
16	1644/1649	Alloaromadendrene	1.16	0.88	1.24	0.84	2.05	0.88
17	1664/1666	Humulene	0.21	0.16	0.20	0.14	0.32	0.14
18	1679/1680	α-Caryophyllene	0.23	0.23	0.30	0.25	0.65	0.20
19	1684/1685	γ-Cadinene	0.18	0.08	0.15	0.10	0.23	0.08
20	1689/1690	Pinocamphone	45.97	20.17	31.91	27.34	22.17	6.09
21	1708/1708	Germacrene D	3.77	2.91	3.82	2.82	5.78	2.76
22	1712/1710	Isocamphopinone	20.10	43.17	32.00	37.62	33.97	54.75
23	1718/1714	γ-Elemene	2.33	2.58	2.72	2.23	3.82	2.40
24	1731/1730	Estragole	0.12	0.10	0.09	0.09	0.10	0.06
25	1743/1740	3-Octen-5-yne, 2,7-dimethyl-, (e)-	0.11	0.15	0.11	0.19	0.11	0.19
26	1755/1756	p-Menth-1-en-8-ol	0.14	0.17	0.16	0.19	0.16	0.22
27	1760/1765	(1r)-(−)-Myrtenal	0.10	0.24	0.21	0.24	0.08	0.23
28	1795/1790	Myrtenol	1.31	1.91	1.85	1.61	1.03	2.01
29	1954/1953	Ledol	0.08	0.07	0.10	0.05	0.16	0.05
30	1997/1990	Elemol	0.32	0.14	0.29	0.11	1.14	0.12
31	2010/2006	Eugenol methyl ether	0.25	0.20	0.23	0.17	0.23	0.16
32	2022/2023	Caryophyllene oxide	0.17	0.19	0.19	0.39	0.34	0.09
33	2136/2126	(−)-Spathulenol	0.28	0.20	0.31	0.14	0.39	0.11
		Total compounds	100	100	100	100	100	100
		Monoterpene hydrocarbons	15.81	22.04	17.72	20.71	16.23	25.75
		Oxygenated monoterpene	70.85	68.26	68.94	69.63	60.87	65.55
		Sesquiterpenes hydrocarbons SH	12.37	9.00	12.37	8.90	20.78	8.27
		Oxygenated sesquiterpenes SO	0.85	0.60	0.89	0.68	2.03	0.36
		Others O	0.12	0.10	0.09	0.09	0.10	0.06

**Table 3:** Inhibition of haemolysis (IH%) and inhibition of protein denaturation (IPD%) and IC50 values of different HOEOS.

mg/mL	1	2	4	8	16	32	64	IC50 mg/mL	1	2	4	8	16	32	64	IC50 mg/mL
Sample	% IH	% IH	% IH	% IH	% IH	% IH	% IH		% IPD	% IPD	% IPD	% IPD	% IPD	% IPD	% IPD	
HORT	−13.04	−7.78	−0.114	5.01	13.28	30.17	63.04	9.09	−1.18	−0.11	0.57	17.69	20.96	23.61	27.39	10.80
HORL	−32.60	−31.88	−14.80	0.61	24.59	42.71	43.81	6.73	−4.82	−1.33	−1.18	19.02	23.12	25.44	29.93	9.70
HOAT	−24.95	−5.56	16.13	23.38	38.92	47.46	58.82	5.87	−3.15	−1.44	5.28	8.20	17.43	20.92	26.13	11.64
HOAL	13.17	15.69	42.18	43.87	55.88	64.16	66.17	4.50	3.04	15.19	27.68	30.40	30.71	32.31	36.70	9.03
HOCT	16.09	28.63	43.89	55.86	62.79	70.82	76.89	3.82	7.12	18.22	33.45	36.71	38.88	41.23	44.52	7.17
HOCL	−19.77	−13.62	−8.01	−6.79	−0.34	13.23	16.72	12.99	−1.77	−1.63	−1.37	−1.33	−0.46	9.57	12.37	24.36

**Table 4:** Bacterial inhibition rates of HOEOs samples against ATCC strains.

Sample mg/mL	*S. pyogenes*	*S. aureus*	*L. monocytogenes*	*B. cereus*	*C. perfringens*	*P. aeruginosa*	*S. flexneri*	*E. coli*	*S typhimurium*	*H. influenzae*
HORT 1	37.25	33.29	11.43	−5.76	6.28	11.23	−4.23	−8.98	12.38	0.58
HORT 2	44.13	36.76	12.72	−4.28	5.57	9.54	0.02	−4.52	16.59	1.49
HORT 4	47.31	37.79	14.38	−3.84	4.83	8.83	1.04	−0.19	19.23	−2.64
HORT 8	51.47	43.77	24.48	4.29	3.27	−0.25	10.26	1.56	28.57	−3.03
HORT 16	53.65	46.66	27.13	6.26	−1.03	−2.65	13.92	5.00	38.68	−3.34
HORT 32	54.60	50.61	31.46	9.36	−3.05	−7.69	23.46	13.26	42.28	−4.00
HORT 64	58.19	55.63	36.17	13.14	−4.80	−11.52	30.65	19.49	50.97	−4.95
HORL 1	47.16	32.54	10.12	−14.69	3.38	3.52	−9.98	22.49	18.16	−4.47
HORL 2	49.98	39.81	12.29	−13.67	3.16	2.67	−7.63	−2.86	20.37	−2.84
HORL 4	51.28	43.67	12.51	−11.05	2.94	1.01	−0.58	1.43	27.12	−1.33
HORL 8	52.35	48.72	25.40	1.02	1.81	−1.39	4.62	5.52	32.22	1.77
HORL 16	53.84	52.78	27.27	3.21	0.86	−6.39	7.29	13.65	40.83	1.01
HORL 32	54.91	55.67	32.76	7.90	0.76	−10.51	19.68	19.10	50.36	0.82
HORL 64	56.85	58.12	37.13	14.55	0.03	−12.65	24.13	24.67	55.78	0.11
HOAT 1	47.02	38.64	7.63	10.66	7.11	9.26	4.82	−0.32	17.82	−5.27
HOAT 2	51.81	41.38	9.86	11.73	6.48	5.25	6.51	1.75	18.72	−4.46
HOAT 4	53.57	46.73	14.19	14.33	5.48	−0.13	12.49	5.59	26.22	−3.70
HOAT 8	54.91	49.62	18.86	21.38	2.46	−2.77	17.62	11.11	28.57	0.73
HOAT 16	59.18	51.65	19.91	28.14	1.59	−6.56	21.11	19.30	36.55	1.66
HOAT 32	60.79	58.73	25.59	31.67	1.65	−10.38	24.58	21.13	42.24	1.96
HOAT 64	66.38	61.27	29.27	34.58	0.73	−16.79	28.72	24.52	48.69	2.89
HOAL 1	52.58	40.01	6.62	−0.90	−5.28	−0.76	8.71	0.32	20.17	−0.79
HOAL 2	54.75	44.73	8.13	12.86	−4.64	−2.65	11.04	3.18	27.07	1.66
HOAL 4	58.04	48.62	13.04	17.77	−3.37	−6.39	16.78	9.94	34.35	1.74
HOAL 8	59.49	51.58	15.78	19.51	1.73	−10.34	21.88	16.83	35.78	2.45
HOAL 16	63.92	57.63	18.28	27.98	3.16	−12.74	27.21	23.13	39.79	2.66
HOAL 32	66.93	63.36	24.29	35.29	4.08	−13.79	33.16	28.63	44.25	3.36
HOAL 64	72.12	69.87	29.34	39.97	5.67	−15.59	38.98	33.12	49.98	4.59
HOCT 1	46.28	20.52	17.59	−5.26	0.89	1.23	−6.54	−4.57	23.22	−7.63
HOCT 2	49.56	22.58	18.39	−3.72	1.46	1.02	−4.15	−2.89	31.47	−5.85
HOCT 4	50.90	24.89	20.20	0.00	2.48	0.84	−1.84	0.97	35.78	−2.31
HOCT 8	52.73	26.09	30.16	4.62	3.29	−1.64	1.45	5.52	36.12	1.60
HOCT 16	54.75	27.88	35.64	11.56	3.29	−4.16	6.71	13.39	42.27	2.47
HOCT 32	58.19	32.54	41.51	14.33	4.29	−7.90	11.81	20.86	44.69	3.29
HOCT 64	60.60	40.68	47.28	24.48	4.83	−11.48	15.27	25.21	47.89	4.02
HOCL 1	50.11	30.49	14.37	−3.68	4.58	4.23	−7.63	0.26	27.00	−8.84
HOCL 2	51.69	33.12	15.68	−1.98	3.68	3.99	−5.98	1.74	32.27	−5.49
HOCL 4	52.62	34.59	16.88	−1.47	2.94	3.19	−2.81	3.90	37.91	−3.05
HOCL 8	53.57	36.28	28.62	6.20	1.60	−0.21	0.19	7.67	45.91	1.85
HOCL 16	54.83	38.52	37.37	13.03	0.82	−1.56	5.91	15.14	54.69	2.58
HOCL 32	58.23	42.51	44.20	17.54	0.15	−5.25	11.14	20.99	58.69	3.43
HOCL 64	61.28	51.55	47.43	26.62	−1.32	−11.39	14.62	23.26	60.25	4.19

**Table 5:** The binding energy between the highly abundant compounds from *Hyssopus* plants and antimicrobial (1KZN) and anti-inflammatory (1NFK, 1VKX) proteins of bacteria.

S/No.	Compounds	Free Binding Energy (Kcal/mol)			Amino Acid Residues Involved in Binding Interaction		
		1KZN	1NFK	1VKX	1KZN	1NFK	1VKX
1	Sabinene	−5.4	−4.4	−5.3	Alkyl: Ile78	Alkyl/Pi-Alkyl: Phe53, Pro68	Alkyl: Leu440, Val442
2	β-Pinene	−4.6	−4.6	−4.9	Alkyl: Ile78	Alkyl: Tyr175	Alkyl: Lys37, Val121, Lys122
3	β-Mircene	−5.1	−4.3	−4.8	Alkyl: Val43, Ala47, Val71, Ile78, Val167	Alkyl/Pi-Alkyl: Lys49, Pro68, Lys77, Tyr79	Alkyl: Phe353, Arg356, Val412, Leu440
4	β-Phellandrene	−5.7	−4.7	−5.9	Alkyl: Val43, Ala47, Val71, Val167	Alkyl: Val120, Ile160, Arg161	Alkyl: Phe353, Arg356
5	Cyclohexene,4-isopropenyl-1-methoxymethoxymethyl	−5.8	−4.9	−5.6	H: Asn46 C-H: Glu50 Alkyl: Ile78	H: Arg154 C-H: Ser110 Alkyl: Val58, Leu140, Val142, Lys146	H: Ser363 Alkyl/Pi-Alkyl: Phe353, Arg356, Val412
6	β-Bourbonene	−6.8	−6.0	−6.0	Alkyl: Ala47, Ile78	Alkyl: Pro68, Lys77	Alkyl: Ala497
7	β-Caryophyllene	−5.9	−6.1	−5.8	Alkyl: Lys189	None	Alkyl: Lys37
8	Alloaromadendrene	−6.4	−6.2	−6.6	Alkyl/Pi-Alkyl: His37, His 38, Ile186	Alkyl: Pro68, Lys77	Alkyl: Arg73
9	Pinocamphone	−5.4	−5.1	−5.0	None	None	None
10	Isopinocamphone	−4.9	−4.9	−5.1	H: Asn46	H: Arg189, Glu190	None
11	γ-Elemene	−6.3	−5.4	−6.1	Alkyl: Ile78	Alkyl: Pro68, Lys77	Pi-Sigma: Tyr538 Alkyl/Pi-Alkyl: Ala545
12	Myrtenol	−4.8	−4.8	−5.4	Alkyl: Ile78	Alkyl: Pro68, Lys77	H: Ser410 Alkyl: Val358, Val442, Leu440, Lys446
13	Elemol	−5.6	−5.8	−5.9	None	Unfavorable Acceptor-Acceptor: Ser78 Alkyl: Pro68	H: His405, Val469, Gln501 Alkyl: Ala497
14	Germacrene D	−6.8	−5.9	−5.8	Alkyl: Ile78	Alkyl: Pro68, Lys77	Alkyl: Pro362, Val412

Green colours: hydrophilic interaction, pink colour, blue colour and red colour: hydrophobic interaction.

## Data Availability

The report of the analysis performed for the samples in the paper can be found at the Interdisciplinary Research Platform (PCI) belonging to the University of Life Sciences “King Michael I of Romania” from Timisoara.

## Supplementary Materials

The following supporting information can be downloaded at www.mdpi.com/article/10.3390/plants13223259/s1, Figure S1: Heatmap of Pearson correlation coefficients for chemical composition.
