# Supplementary material for: Chemical and Biological Properties of Different Romanian Populations of Hyssopus officinalis Correlated via Molecular Docking
Source: Plants (Basel). 2024 Nov 20;13(22):3259. doi: 10.3390/plants13223259 (PMC11598396; doi:10.3390/plants13223259)
Supplement: Supplementary file 1 [file plants-13-03259-s001.zip › plants-3257319-supplementary.pdf]

Supplemmentary files - Heatmap of Pearson correlation coefficients for chemical composition.

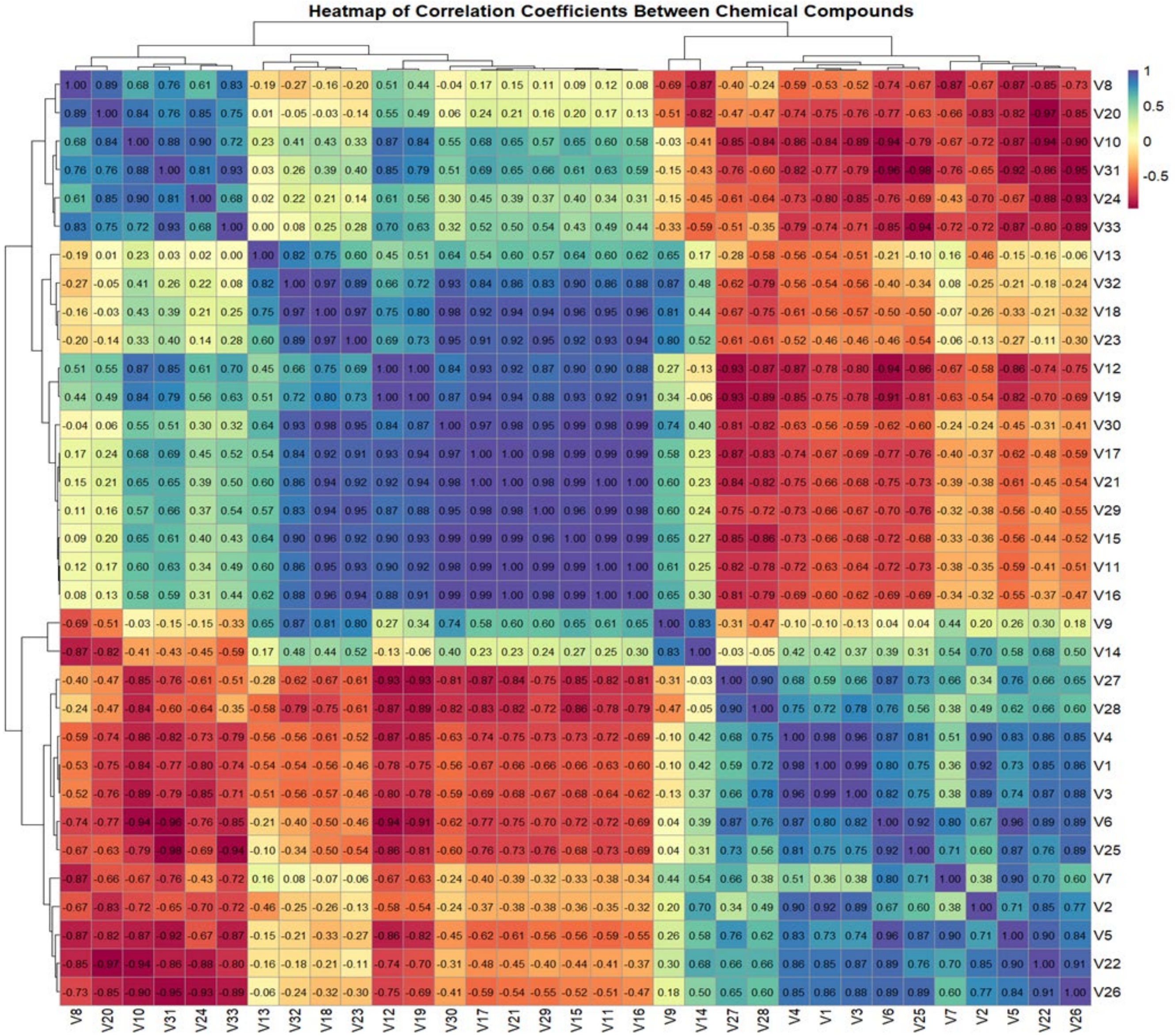

Note: V1: α-pinene, V2: α-Phellandrene, V3: β-pinene, V4: Sabinene, V5: β-Myrcene, V6: (-)-Limonene, V7: β-Phellandrene, V8: β-cis-Ocimen, V9: Eucalyptol, V10: cyclohexene, 4-isopropenyl-1-methoxymethoxymethyl-, V11: α-Gurjunene, V12: β-Bourbonene, V13: Linalool, V14: 3-Thujanone, V15: Caryophyllene, V16: Alloaromadendrene, V17: Humulen-(v1), V18: α-Caryophyllene, V19: γ-Cadinene, V20: Pinocamphone, V21: Germacrene d, V22: Isocamphopinone, V23: γ-Elemene, V24: Estragole, V25: 3-Octen-5-yne, 2,7-dimethyl-, (e)-, V26: p-menth-1-en-8-ol, V27: (1r)-(-)-Mirtenal, V28: Myrtenol, V29: Ledol, V30: Elemol, V31: Eugenol methyl ether, V32: Caryophyllene oxide, V33: (-)-Spathulenol.

Figure S1. Heatmap of Pearson correlation coefficients for chemical composition.
